# Observational study of ground-level ozone and climatic factors in Craiova, Romania, based on one-year high-resolution data

**DOI:** 10.1038/s41598-024-77989-0

**Published:** 2024-11-05

**Authors:** Hasan Yildizhan, Mihaela Tinca Udriștioiu, Tugce Pekdogan, Arman Ameen

**Affiliations:** 1 Engineering Faculty, Energy Systems Engineering, Adana Alparslan Türkeş Science and Technology University, Adana, 46278 Turkey; 2 Clean Energy Processes (CEP) Laboratory, Department of Chemical Engineering, Imperial College London, London, SW7 2AZ UK; 3 Faculty of Science, Physics Department, University of Craiova, 13 A.I. Cuza Street, Craiova, 200585 Romania; 4 Department of Architecture, Faculty of Architecture and Design, Adana Alparslan Türkeş Science and Technology University, Adana, 46278 Turkey; 5 Department of Building Engineering, Energy Systems and Sustainability Science, University of Gävle, Gävle, 801 76 Sweden

**Keywords:** Monitoring system, Ozone concentration, Meteorology, Natural and anthropocentric factors, Python, SPSS, Multiple linear regression, Ozone weekend effect, Mid-sized city, Environmental sciences, Environmental chemistry, Environmental impact, Public health

## Abstract

Air pollution is a multifaceted issue affecting people’s health, environment, and biodiversity. Gaining comprehension of the interactions between natural and anthropocentric pollutant concentrations and local climate is challenging. This study aims to address the following two questions: (1) What is the influential mechanism of climatic and anthropogenic factors on the ground-level ozone (O_3_) concentrations in an urban environment during different seasons? (2) Can the ozone weekend effect be observed in a medium-sized city like Craiova, and under which conditions? In order to answer these questions, ozone interactions with meteorological parameters (temperature, pressure, relative humidity) and pollutant concentrations (particulate matter, carbon dioxide, volatile organic compounds, formaldehyde, nitrogen dioxide, nitric oxide and carbon monoxide) is evaluated based on a one-year dataset given by a low-cost sensor and one-year dataset provided by the National Environment Agency. Using two statistical analysis programs, Python and SPSS, a good understanding of the correlations between these variables and ozone concentration is obtained. The SPSS analysis underscores the significant impact of three meteorological factors and nine other pollutants on the ozone level. A positive correlation is noticed in the summer when sunlight is intense and photochemical reactions are elevated. The relationship between temperature and ozone concentration is strong and positive, as confirmed by Spearman’s rho correlation coefficient (*r* = 0.880). A significant negative correlation is found between relative humidity and ozone (*r* = -0.590). Moreover, the analysis shows that particulate matter concentrations exhibit a significant negative correlation with ozone (*r* ≈ -0.542), indicating that higher particulate matter concentrations reduce ozone levels. Volatile organic compounds show a significant negative correlation with ozone (*r* = -0.156). A negative relationship between ozone and carbon dioxide (*r* = -0.343), indicates that elevated carbon dioxide levels might also suppress ozone concentrations. A significant positive correlation between nitrogen dioxide and ozone (*r* = 0.060), highlights the role of nitrogen dioxide in the production of ozone through photochemical reactions. However, nitric oxide shows a negative correlation with ozone (*r* = -0.055) due to its role in ozone formation. Carbon monoxide has no statistically significant effect on ozone concentration. To observe the differences between weekdays and weekends, T-Test was used. Even though significant differences were observed in temperature, humidity, carbon dioxide, volatile organic compounds, nitrogen dioxide, nitric oxide and carbon monoxide levels between weekdays and weekends, the T-Test did not highlight a significant weekend ozone effect in a mid-sized city as Craiova. Using Python, the daily values were calculated and compared with the limit values recommended by the World Health Organization (WHO) and European Environment Agency (EEA). The WHO O_3_ recommended levels were exceeded for 13 times in one year. This study offers a comprehensive understanding of ozone pollution in a mid-sized city as Craiova, serving as a valuable reference for local decision-makers. It provides critical insights into the seasonal dynamics of ozone levels, emphasizing the significant role of temperature in ozone formation and the complex interactions between various pollutants and meteorological factors.

**Supplementary Information:**

The online version contains supplementary material available at 10.1038/s41598-024-77989-0.

## Introduction

Ground-level ozone (O_3_), also known as tropospheric ozone, represents a significant air pollutant formed in photochemical reactions between volatile organic compounds (VOC) and nitrogen oxides (NO_x_) during hot and sunny days when the sunlight produces the amount of energy that is necessary for such chemical reactions to occur^1^. A study conducted in China has revealed that the ozone formation process is more efficient in urban areas where air pollution is higher than in rural areas^2^. Ground-level ozone represents a significant component of the smog in urban areas^3^. High ozone concentration threatens indiscriminately human health^4^, the environment, crops, and biodiversity^5^. Exposure to high ozone concentration is associated with respiratory issues^6^, cardiovascular events^7^, hospital admissions and mortality^8^. These findings underscore the urgent need to address the issue of ozone concentration.

The ground-level ozone concentration can be potentially reduced by implementing local strategies such as controlling emission vehicles inside of the cities by limiting car traffic in city centres and imposing high parking charges^9^, making ring roads around cities to avoid urban high traffic^10^, wetting down construction sites with water, vacuum, sweeping and wetting paved surfaces and roads^11^, using filters to control thermal power plant emissions, enforcing industrial facilities to reduce the emissions of VOC and NO_x_through the use of some new technologies like catalytic converters and scrubbers^12^, regulating the use of consumer products such as paints, solvents and cleaners to limit VOC emissions^13^, replacing pollutant technologies with cleaner ones^14^, better education in using more public transportation^15^, developing public awareness about the importance of clean air for human health^16^. These highlight the potential of local strategies in combating air pollution, instilling hope in the citizens.

The first step in controlling ground-level ozone concentrations is to understand locally the interaction between all factors that influence ozone production. Meteorological parameters such as temperature, sunlight, wind speed and direction, atmospheric stability, and humidity affect atmospheric chemistry and transport^17^. Studies on ozone and fine particulate matter concentrations conducted in the USA^18,19^ show that global climate change and emission control strategies significantly impact air pollutants. The relation between particulate matter concentrations (PM_1_, PM_2.5_, PM_10_)^20^ and ozone level is complex. It can vary depending on factors such as the composition of PM, atmospheric conditions, and local emissions sources. PM concentrations can lead to the production of ozone precursors (VOC and NO_x_) or can produce a scavenging effect by absorbing it. The second step is to choose the most appropriate measures that the decision-makers^21^ can implement locally, based on forecasting ground-level ozone concentrations, considering the types of sources of air pollutants and the financial possibilities to improve air quality for citizens’ benefit.

Table 1 thoroughly examines the literature concerning ozone pollution and its repercussions on human health, indoor air quality, and the environment. The table categorizes studies on the impact of outdoor air pollution on indoor air quality, the potential health risks associated with ozone pollution, and methods for addressing these risks. Research addressing the correlation between ozone and various health issues, such as asthma, eye diseases, respiratory infections, and depression, underscores the broad range of effects of ozone pollution on human health. Furthermore, investigations on innovative approaches to manage and reduce indoor ozone pollution provide insight into technological advancements in this area.

**Table 1:** Literature review of ozone pollution.

Ref No	Year	Country	Place	Pollutants	Main Topic	Summary
Chiritescu et al^22^.	2024	Romania	Urban areas	PM_10_, PM_2.5_, NO_2_, CO, SO_2_, O3	Based on the dataset provided by the Romanian National Network of Air Quality Monitoring	Emphasize the impact of lockdown on air pollution in 33 cities across
El Mghouchi et al^23^.	2024	Romania	Urban area, Craiova	O_3_, PM_10_, PM_2.5_, PM_1_, CO_2_, VOC, CH_2_O, RH, T, P	Forecasting ground ozone levels and air quality based on the Meta Hybrid Deep Learning Model	O_3_ concentration is primarily influenced by meteorological variables such as temperature, sunshine duration and relative humidity and secondarily by air pollution indicators including VOC, PM and CO_2_
El Mghouchi et al^24^.	2024	Romania	Urban area, Craiova	PM_10_, PM_2.5_, PM_1_, VOC, CO_2_, CH_2_O, T, RH, P,	Air quality predictions and multivariate modelling via five hybrid machine learning models	PM_10_ concentrations exhibited a notable correlation with PM_2.5_ concentrations and a moderate correlation with PM_1_. PM concentrations were not strongly related to CO_2_ and VOC, and these last variables should be combined with another meteorological variable to enhance the prediction accuracy.
Petrus et al^25^.	2024	Romania	Urban area	C_2_H_4_, C_6_H_6,_ C₆H₅CH₃, and O_3_	A detector based on laser photoacoustic spectroscopy was utilised to monitor pollutants in Magurele, Romania	Diurnal patterns were noticed for C_2_H_4_, C_6_H_6_, and C₆H₅CH₃, which peaked during the early hours of the day. O_3_ concentrations peaked in the evening.
Link et al^26^.	2023	-	Residential buildings	NO_x_, VOC	Impact of Outdoor NO_x_ Pollution on Indoor Air	Studied how outdoor NO_x_ affects indoor air chemistry through ventilation in residential buildings.
Udristioiu et al^27^.	2023	Romania	Urban area, Craiova	PM_10_, PM_2.5_, PM_1_, CO_2_, VOC, O_3_, CH_2_O, RH, T, P	Prediction, modelling, and forecasting of PM and AQI using hybrid machine learning	Based on a combination of hybrid models, such as Input Variable Selection, Machine Learning, and a regression method, the daily concentrations of PM_1_, PM_2.5_, PM_10_, and AQI were predicted, modelled, and forecast.
Vasile et al^28^.	2023	-	Residential buildings	O_3_, PM2._5_, CH_2_O, CO_2_	Influence of Ventilation on Indoor Air Quality	Discussed the impact of ventilation on indoor air quality, focusing on ozone.
Zoran et al^29^.	2022	Romania	Metropolitan region Bucharest	^222^Rn, PM_2.5_, PM_10_, NO_2_, O_3_, SO_2_, CO	Exposure to air pollutants (including radon) and climate factors impacted all five COVID-19 waves	Studied the impact of environmental cumulative effects of anthropogenic air pollution and climate variability on COVID-19 multi-wave severity in Bucharest
Jing and Wang^30^	2022	China	Residential buildings	O_3_	Indoor Ozone Pollution Based on Outdoor Air Pollution	Highlighted health risks of indoor ozone pollution influenced by outdoor air.
Piccirillo et al^31^.	2022	-	-	O_3_	Air Pollution as a Risk Factor for Cardioinhibitory Response	Linked high ozone concentrations to a slightly increased risk of cardioinhibitory response.
Vasiliauskienė and Vasilis Vasiliauskas^32^	2022	-	Office/lab environment	O_3_	Indoor Air Pollution from Copiers and Printers	Examined indoor air pollution levels during copying processes and their effects.
Peeters et al^33^.	2022	Belgium	-	O_3_, PM_2.5_	Air Pollution and Symptom Severity in CRS Patients	Examined the impact of air pollution on symptom severity in CRS patients.
Nistor et al^34^.	2021	Romania	Urban areas	O_3_	The maximum values of O_3_ concentration are recorded in April-May (when UV has a maximum intensity)	O_3_ concentrations (2009–2020) were lower than the threshold value (> 180 µg/m3) in the North-East of Romania
Iordache et al^35^.	2021	Romania	Urban area	O_3_	Real-time monitoring of ozone concentration variation within urban environment	Analysis of yearly and daily variation of the ozone concentration in Bucharest
Lannuque et al^36^.	2021	-	-		Modelling of OH and HO2 Radicals in Air Quality Models	Discussed the importance of accurately modelling radicals for understanding air quality.
Dores et al^37^.	2021	Canada	-		Outdoor Air Pollution and Depression	Investigated the relationship between outdoor air pollution, including ozone and depression.
Morici et al^38^.	2020	-	Urban areas	O_3_, secondary organic aerosols	Effects of Ozone Exposure During Exercise	Highlighted adverse effects of ozone exposure on pulmonary function during exercise.
Mendoza et al^39^.	2020	-	Schools	O_3_, SO_2_, NO_2_	Impact of Ozone Exposure on School Absences	Highlighted the association between ozone exposure and school absences.
Han et al^40^.	2020	China	-	O_3_	Outdoor Air Pollution Concentrations and COVID-19 Infections	Explored the impact of outdoor air pollution on COVID-19 infections in China.
Ye et al^41^.	2020	China	Office/lab environment	O_3_	Ozone Deposition on Indoor Materials and VOC Emissions	Studied ozone deposition on various indoor materials and resulting VOC emissions.
Urbina Guerrero et al^42^.	2020	Brazil	-	O_3_	Nocturnal Ozone Increase in São Paulo	Studied the formation of nocturnal ozone peaks in São Paulo and their health impacts.
Geddam et al^43^.	2020	India	-	O_3_, VOC	Ground-Level Ozone Concentration and Its Impact	Evaluated the 8-hour average ground-level ozone concentration in Visakhapatnam.
Emetere et al^44^.	2019	Cameroon	-	O_3_	Outdoor Air Pollution Sources and Trends	Analysed aerosol loading in Kumbo, Cameroon, emphasising the need for data-driven research.
Sweileh et al^45^.	2019	-	-	O_3_	Bibliometric Analysis on the Impact of Outdoor Air Pollution on Respiratory Health	Conducted a bibliometric analysis of the research trends concerning outdoor air pollution and respiratory health.
Yang et al^46^.	2018	China	Student dormitories	O_3_, other outdoor pollutants	Ventilation and Air Quality in Student Dormitories	Delved into ventilation and air quality in student dormitories, focusing on ozone levels.
Havet et al^47^.	2018	France	-	O_3_, PM_1_, PM_2.5_, PM_10_	Outdoor Air Pollution and Asthma in Adults	Investigated the link between outdoor air pollution, especially ozone, and asthma in adults.
Kalimeri et al^48^.	2017	Greece	-	O_3_	Personal Exposure to Ozone in Athens	Monitored personal ozone levels for individuals in Athens, focusing on indoor and outdoor exposure.
Hwang et al^49^.	2016	South Korea	-	O_3_	Outdoor Air Pollution and Dry Eye Disease	Explored ozone’s potential significance in the correlation with dry eye disease in South Korea.
Singer et al^50^.	2016	USA	Residential buildings	O_3_	Filtration and Ventilation Systems Performance	Evaluated the performance of different filtration and ventilation systems in a modern house.
Adam-Poupart et al^51^.	2015	Canada	-	O_3_	Outdoor Ozone and Respiratory Diseases Among Workers	Examined the association between outdoor ozone levels and respiratory diseases among workers.
Xiaobing et al^52^.	2015	-	-	O_3_, PM_2.5_	Indoor Ozone Pollution and Purification Technologies	Investigated indoor ozone pollution and potential purification technologies.

A statistical analysis is performed using IBM SPSS Statistics^53^version 26 to understand the overall distribution of variables locally. Based on Python, the number of days exceeding the limit value is evaluated according to the recommendations of the World Health Organization (WHO)^54^‎ and European Union air quality standards established in Directive 2008/50/EC, Air Quality^55^. The WHO has recommended that ozone concentrations should not exceed 60 µg/m^3^ in case of long-term exposure. However, the EU Air Quality Directive sets a threshold value of 120 µg/m^3^. A multiple linear regression model is developed to understand how the chosen independent variables influence ground-level ozone concentrations. As an advantage of the statistical approach, ANOVA analysis tests the significance of the proposed model. The R-square value shows how much the independent variables explained the total variance in ozone. The regression coefficients of each variable and the statistical significance of these coefficients (t-test and p-values) are evaluated. Some of the limitations of this research are that only one sensor was used to measure ozone concentration, and this sensor cannot measure wind speed and direction, sun brightness, NO, NO_2_ and CO concentrations. To counteract some of the limitations of this study, NO, NO_2_ and CO data were downloaded from the nearest local environmental agency station to the monitor A3. Craiova has an independent network of air quality monitoring sensors, but most measure PM concentrations and meteorological parameters. One strength is that the monitor A3, which can measure ozone concentration, was in its first year of operation when it took measurements and was calibrated before measurements began.

The results of this study contribute to the current state of research by applying a comprehensive analysis integrating environmental and meteorological factors over one year in a Romanian mid-sized city (Craiova). Unlike previous studies that may focus on single variables or different geographic regions, our research employs a robust dual-method approach using Python version 3.12.0^56^ and SPSS to evaluate correlations, exceedance thresholds and weekend effect and to test the significance of the proposed models. Also, this study shows that the weekend ozone effect was less significant in a Craiova than in large cities during the pandemic. During the weekend, NO_x_ concentrations decreased, and VOC increased. The level of the ozone weekend effect due to the reduction of NO_x_ emissions is not as effective as when there is a simultaneous reduction of NO_x_ and VOC concentrations. Another explanation could be given by the restrictions imposed by the Romanian authorities during the COVID-19 pandemic, which were less severe during the studied period (December 10, 2020 - December 9, 2021) than the state of emergency (March 16, 2020 - May 15, 2020). The novel elements of this study are the following: (1) Using advanced statistical methods in processing high-density sensor data, our study offers new statistical insights into the ground-ozone concentration level and emphasises an insignificant ozone weekend effect in Craiova, a mid-size Romanian city; (2) A combination of two datasets with a high granulation was used in this work. The datasets were provided by a low-cost A3 monitoring system belonging to a community sensor network and an AQ monitoring station belonging to the county environmental agency. This emphasises the added value of the complementarity between official and independent sensor networks; (3) The findings offer a new regional perspective on air quality management. In the future, local reductions of traffic and industrial activities during ozone pollution episodes may not be sufficient to reduce ozone levels, as local strategies in case that will be necessary. Ozone concentrations are currently within the WHO guidelines and EEA limits. Variations in meteorological parameters (temperature, relative humidity under solar radiation, wind speed and direction) play a vital role in the intensity of changes in the gaseous precursors of ozone. Thus, they represent a source of ground-level ozone generation locally March 16, 2020 - May 15, 2020.

This study is structured as follows: Sect. 2 describes sensor location, climate features of the geographic area, datasets, and data analysis. Section 3 gives an overview of the steps performed using descriptive analysis, correlation analysis, ozone thresholds, and multiple linear regression analysis and points to the results of each analysis. Additionally, the results are compared with those of other studies. Section 4 concludes the paper with a summary of the main findings.

## Materials and methods

### Location

**Fig. 1:**
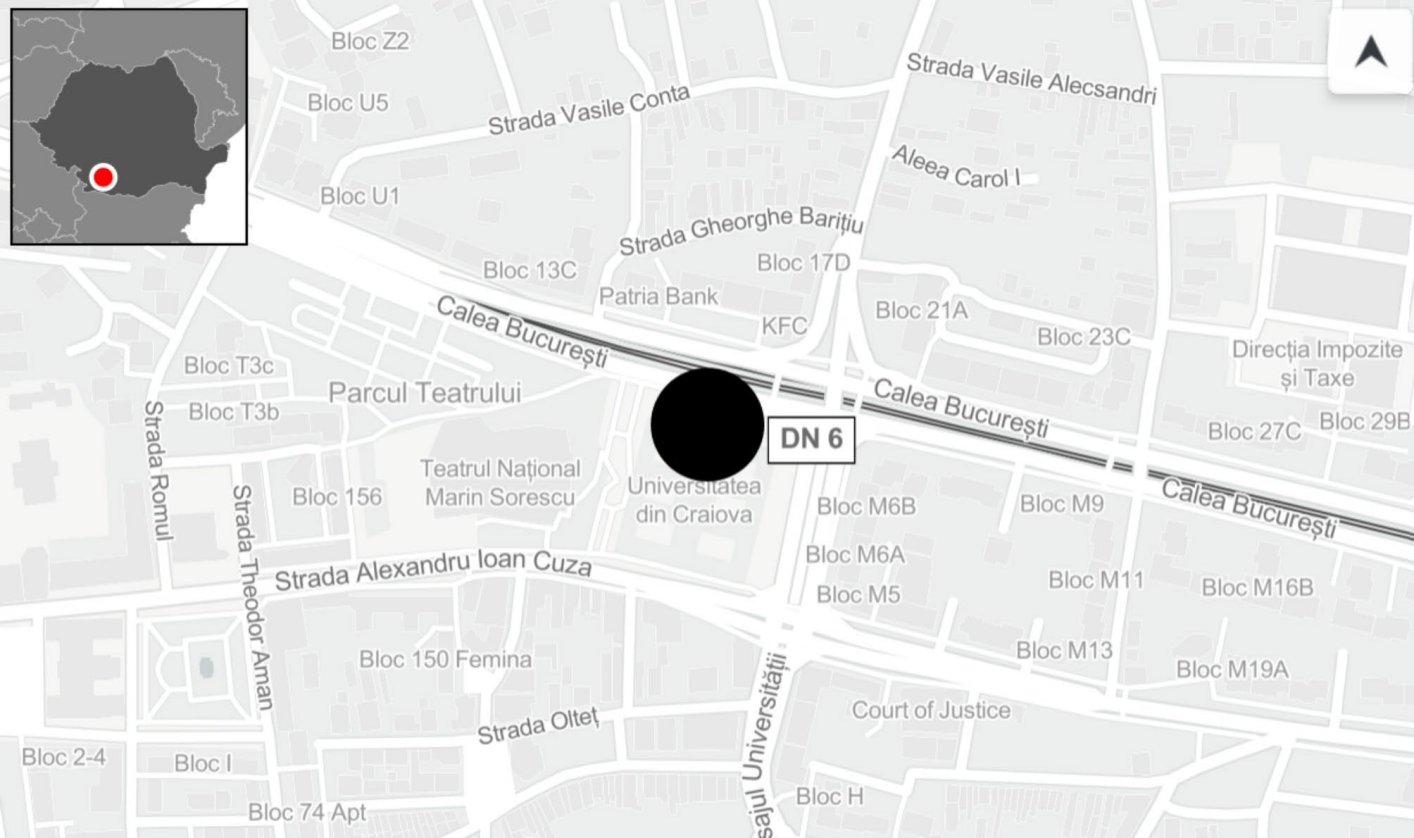
Sensor location in Craiova, Romania^57^.

As is shown in Fig. 1(created using Mapcreator^57^), the sensor ID 820002C3 is in the centre of Craiova, one of the southwestern cities of Romania, and has a fixed location (near a busy traffic intersection). From an economic point of view, Craiova is the heart of the southwestern part of Romania and the sixth city in Romania in terms of population (243 765 inhabitants, according to the 2022 census, National Institute of Statistics, Romania). The population density in Craiova is over 3.700 loc/km^2^. It has a surface of 81.41 km^2^. The city is continuously developing, with a busy traffic infrastructure, many construction sites, small firms and companies and an old heating centralised system^58^. Craiova still has an essential green surface (parks) of 196 hectares^59^.

Craiova is a mid-sized city with high traffic, a busy transport infrastructure, and many old cars. The city’s northern ring road opened in 2006, and the southern belt will be operational in 2025. In 2021, the Romanian Automobile Manufacturers and Importers Association reported only 121,208 new cars and 395,759 s-hand car registrations, while 420,755 cars were produced in Romania by the two major manufacturers (Car production in Romania in 2021). Although the Romanian government has developed the “Rabla” program to remove old cars from the market, many second-hand cars are registered yearly in Romania. Craiova respects the national trends regarding the new and second-hand car registrations ratio. The Sustainable Urban Mobility Plan for the Craiova growth pole shows the traffic analysis on October 25, 2021, using a side radar measuring system located on Carol I Boulevard, which is at 200 m distance from the location of monitor system A3. The results showed that traffic reached its maximum on this boulevard at 17:30 (309 cars). High traffic values are recorded between 7:00 am (290 cars) and 8:00 am (205 cars) and from 12:30 pm (200 cars) to 6:30 pm (215 cars).

In 1990, Craiova had almost 80,000 apartments connected to the centralised system (supported by a coal-fired power plant); in 2021, there were just over 40,000. Local strategies include developing the gas supply network in residential areas, but some still use coal, vegetable waste, or wood heating. In recent years, the burning of garden waste, plastics and tyres in residential areas has significantly decreased due to the fines issued by the environmental guard and the numerous educational projects developed at the local level (Clear Air Craiova, Clear Air Oltenia, Prevent).

The climate in Craiova is transitional temperate continental with Mediterranean influences, with mild winters and higher rainfall (especially during autumn). 300 h is the maximum sunshine duration in July, and 250 h in June (2200 yearly average sun hours). The highest level of UV radiation is recorded in June and July when the UV index is 9, followed by May and August when it is 8. The highest monthly mean number of wind speed days exceeding 16 m/s is recorded during Spring (March and April). The wind primarily comes from the NE-E and W. A local dry wind, Austru, blows all seasons from the west-south-west and south of the Oltenia region. It brings heat and drought during summer and amplifies the frost during Winter^60,61^.

### Dataset description

The first dataset was provided by an automatic monitoring system with the identification number ID 820002C3, starting on December 10, 2020, and ending on December 9, 2021. The location of this monitor is on the outside wall of the University of Craiova, facing a busy intersection (Calea Bucuresti Street and Carol I Avenue). The system is a uRADmonitor A3 model, made in Romania (Real-time Environmental Monitoring, n.d.), calibrated before the sale, and certified by three internationally accredited laboratories from Romania (ECOIND), France (AIRPARIF) and the USA (AQSPEC). The evaluation reports are made public on the manufacturer’s site, together with the technical specifications of the monitoring system^62,63^.

**Fig. 2:**
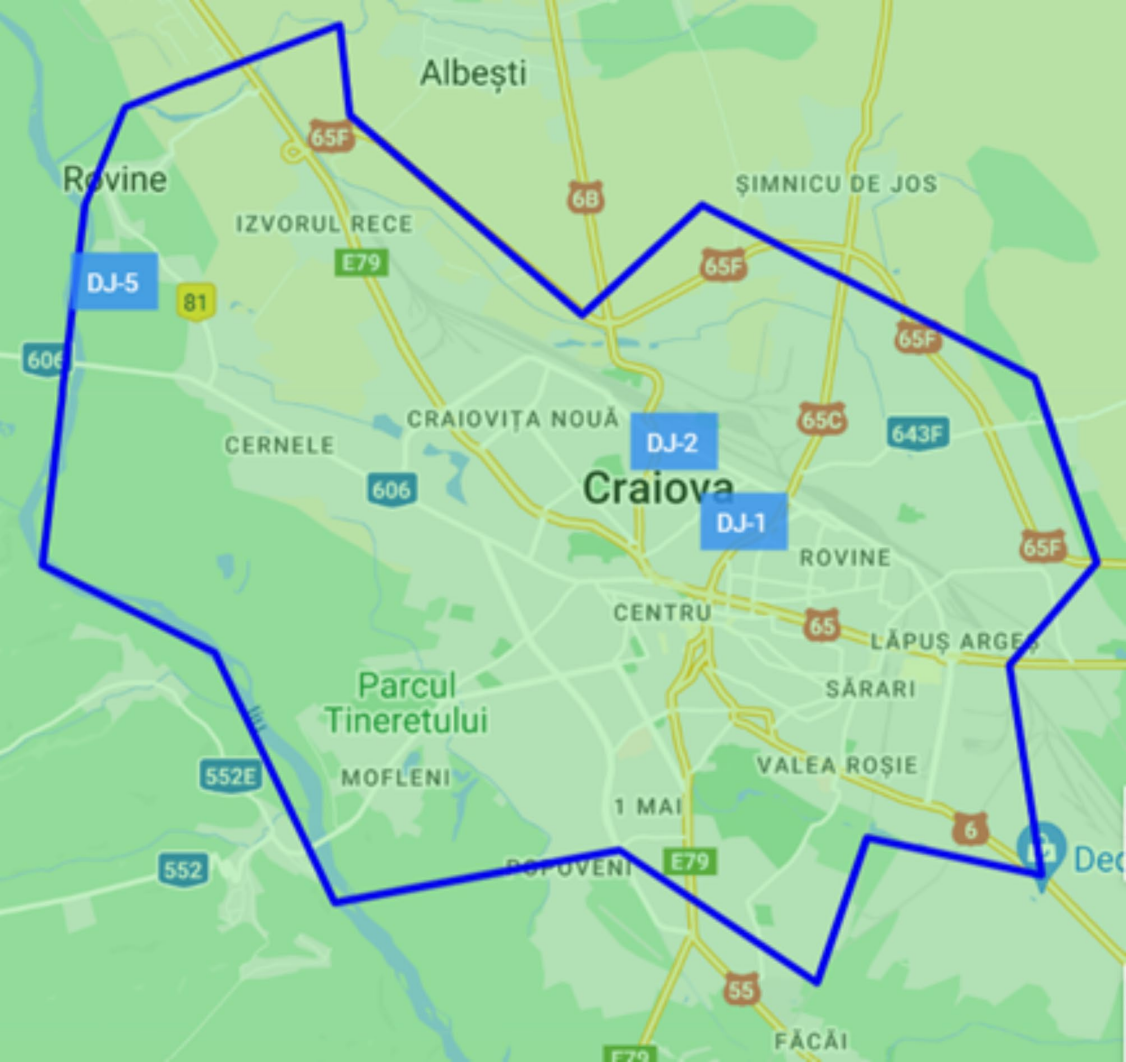
Official stations DJ1, DJ2 and DJ5 that belong to the Romanian National Air Quality Monitoring Network^66^.

The monitor A3 (Lat. 44.3194 º, Long. 23.8011º, Alt. 120 m) is part of the network of sensors Clear Air Craiova, made in the framework of academic-community cooperation, for educational and research purposes^64^. This academic network contains 11 monitors, from which 10 are model Smoggie PM (measure six parameters: three meteorological parameters and three PM concentrations), and one is model uRADMonitor A3 (additionally, it measures ground ozone (O_3_), formaldehyde (CH_2_O), carbon dioxide (CO_2_), volatile organic compounds (VOC)). Clear Air Craiova network is part of a more extensive network (www.uradmonitor.ro). Clear Air Craiova network, independent of the National Air Quality Monitoring Network, appeared in 2020 under poor communication from the County Environment Agency and a lack of data during air pollution episodes^65^. The National Air Quality Monitoring Network (www.calitateaer.ro) has three stations in Craiova (DJ1-a traffic station, DJ2- an urban background station, and DJ5- a mixt industrial and traffic station, see Fig. 2).

The monitor A3 measures, at every minute, eleven parameters: temperature T, air pressure P and relative humidity RH, PM_1_, PM_2.5_ and PM_10_, VOC, CH_2_O, CO_2_, noise level and O_3_ concentrations. The life span for the electrochemical sensors that measure O_3_ and CH_2_O concentrations is two years. 2020–2021 was the first year of operation of the sensor ID 820002C3. PM concentrations are measured using the laser scattering method, CO_2_ is measured by a nondispersive infrared sensor, a metal oxide sensor measures VOC, and micro-electromechanical systems measure meteorological parameters. More information about the technical specifications of the monitor A3 is included in Supplementary Annex A.

The second hourly dataset related to NO_2_, NO and CO was downloaded from the National Air Quality Monitoring Network platform for the same time interval. The last three parameters are very important in the process of O_3_ production. The station DJ1 (Lat. 44.318611º, Long. 23.80612º, Alt. 118 m) is the closest to the location of the monitor A3 (200 m). The second dataset was needed to obtain information about ozone precursors, which the first monitor, A3, cannot provide.

### Data analysis

According to the Air quality maintenance plan in Dolj County (where Craiova is located) 2020–2025, the sources of PM_2.5_ and PM_10_ pollution are 97% surface sources (heating buildings with solid fuel (coal, wood, biomass) or gas, gas stations, landfills, raw material/fuel depots), 2.7% mobile sources (road, rail, and air transport), and 0.3% stationary sources (sources of direct emissions; belong to the industrial sector, including the energy sector). PM concentrations are highest in winter when industrial and domestic combustion processes for heating are decisive. The main sources of NO_x_ are: >77.4% from mobile sources (road, rail and air transport), 19.6% from surface sources (electricity and heat production and supply), almost 3% from stationary sources (treatment and processing of raw materials for the manufacture of food and feed). For CO, almost 88% comes from surface sources (household heating, illegal burning of tyres and plastic waste in backyards), 12% from mobile sources (transport), and 0.31% from stationary sources (combustion of fuel in installations, disposal of hazardous waste). For VOC, almost 56% is from household air emissions, 44% from industry (surface treatment of metals, objects, or products using organic solvents), and transport.

The relationships between O_3_ concentrations, other air quality indicators, and meteorological variables are investigated based on measurements made over a year in Craiova, Romania. The statistical analysis evaluates meteorological parameters (T, P, RH), particulate matter concentrations (PM_1_, PM_2.5_, PM_10_), CO_2_, VOC, CH_2_O, NO_2_, NO, CO and O_3_ levels.

In this study, statistical analyses are obtained using IBM SPSS Statistics version 26^53,67^. As a first step, basic statistical summaries of each variable are obtained using the descriptive statistical analysis method. Basic statistical summaries of each variable were obtained, including mean, median, standard deviation, minimum, and maximum values, to understand the overall distribution of the data. These statistics represent an essential step in gaining knowledge about the overall distribution of variables. Here, 12-month data is divided into four seasons and analysed based on the three-month data average. Both parametric and nonparametric tests were utilised to ensure the robustness of the findings. To address any concerns regarding the normality of the data, Spearman’s rho, a nonparametric correlation test, was also employed. The correlation coefficients^68^determine the strength and direction of the relationships between variables. The resulting correlation matrix is constructed to reveal which air quality parameters have strong positive or negative relationships with each other and which show insignificant relationships. The study investigates the suitability of the data for these limit value calculations, primarily based on the information regarding the limit values of WHO. Python is used in these calculations^69,70^. With the code created in Python, the number of days exceeding the limit value in a year is found. An MLR model is developed to understand ozone concentration factors with these calculations, including air quality and meteorological indicators selected as independent variables. A regression model is used to determine the variables’ relative importance and direction of influence on ozone levels. ANOVA analysis is performed to test the significance of the model, and the R-square value shows how much the independent variables explained the total variance of ozone. Additionally, the regression coefficients of each variable and the statistical significance of these coefficients (t-test and p-values) are evaluated.

## Results and discussion

### Descriptive analysis

Table 2 shows the data collected through fieldwork in this study and provides a comprehensive overview showing various measured environmental parameters, including T, RH, PM_1_, PM_2.5_, PM_10_, CO_2_, O_3_, VOC, CH_2_O, NO_2_, NO, and CO. This dataset carries 8308 valid observations for each parameter. Here, the average, standard deviation, median, and quartiles (25%, 50% and 75%) of the measurement results taken for 12 months are presented. The data in Table 2 represent the seasonal averages of O_3_ levels, calculated from daily measurements over the study period.

In the winter season, the average temperature was recorded as 3.2 °C, and the standard deviation was 4.3 °C. The mean of ozone levels in winter is 22.3 µg/m³, and the standard deviation is 2.9 µg/m³, which reflects the lower photochemical activity during this period. The average relative humidity was 80.7%, and the pressure was 100,384.6 Pa. The particulate matter levels were recorded as 29.9 µg/m³ for PM2.5 and 33.6 µg/m³ for PM10. The CO_2_ concentration averaged 563.4 ppm, CO levels were 0.9 µg/m³ and VOC levels were 153,721.2 ppb. The mean CH_2_O concentration was 14.1 µg/m³. Additionally, NO_2_ concentrations were 20.6 µg/m³ and NO concentrations were 4.9 µg/m³.

In the spring season, the average temperature was 10.5 °C, and the standard deviation was 6.3 °C. The mean ozone level in spring is 29.6 µg/m³, and the standard deviation is 6.5 µg/m³, which may indicate increased sunlight and photochemical activity. The average relative humidity was 74.5%, and the pressure was 100,354.5 Pa. Particulate matter levels were recorded as 13.7 µg/m³ for PM2.5 and 15.2 µg/m³ for PM10. The CO_2_ concentration averaged 572.6 ppm, CO levels were 0.1 µg/m³ and VOC levels were 160,503.8 ppb. The mean CH_2_O concentration was 14.1 µg/m³. Additionally, NO_2_ concentrations were 11.5 µg/m³ and NO concentrations were 3.5 µg/m³.

In the summer months, increasing temperatures and ozone levels were observed. The average temperature was 23.9 °C, and the standard deviation was 5.6 °C. The mean ozone level in summer is 44.1 µg/m³, and the standard deviation is 10.1 µg/m³, suggesting that ozone may increase during the summer months due to photochemical formation. The average relative humidity was 72.1%, and the air pressure was 100,120.9 Pa. Particulate matter levels were 7.6 µg/m³ for PM2.5 and 8.1 µg/m³ for PM10. The CO_2_ concentration was 544.9 ppm, CO levels were 0.0 µg/m³ and VOC levels were 167,129.2 ppb. The mean CH_2_O concentration was 14.1 µg/m³. NO_2_ concentrations were 6.2 µg/m³ and NO concentrations were 2.7 µg/m³.

In the autumn season, temperatures and air pressure drop, and humidity levels rise. The average temperature was 11.5 °C, and the relative humidity was 78.7%. The pressure was 100,749.2 Pa. Particulate matter levels were recorded as 18.8 µg/m³ for PM2.5 and 20.9 µg/m³ for PM10. The CO_2_ concentration was 593.9 ppm, CO levels were 0.4 µg/m³ and VOC levels were 188,625.0 ppb. The mean CH_2_O concentration was 14.1 µg/m³. Additionally, NO_2_ concentrations were 9.1 µg/m³ and NO concentrations were 4.0 µg/m³.

**Table 2:** Descriptive analysis of the data.

			T (°C)	P (Pa)	RH (%)	PM_1_ (µg/m³)	PM_2.5_ (µg/m³)	PM_10_ (µg/m³)	CO_2_ (ppm)	O_3_ (µg/m³)	VOC	CH_2_O (µg/m³)	NO_2_ (µg/m³)	NO (µg/m³)	CO (µg/m³)
											(ppb)				
Winter	Mean		3.2	100384.6	80.7	23.9	29.9	33.6	563.4	22.3	153721.2	14.1	20.6	4.9	0.9
	Median		3.1	100324.0	81.8	18.0	22.8	25.4	558.7	21.2	145944.3	14.1	17.3	3.0	0.2
	Percentiles	25	0.8	99685.0	76.9	10.8	13.9	15.3	517.6	20.2	129644.2	14.0	11.5	2.0	0.1
		50	3.1	100324.0	81.8	18.0	22.8	25.4	558.7	21.2	145944.3	14.1	17.3	3.0	0.2
		75	5.8	101006.3	85.4	29.5	36.6	41.3	604.0	23.3	174174.8	14.3	26.1	5.3	0.4
	Std. Dev.		4.3	884.4	6.5	20.9	25.6	29.2	64.9	2.9	36895.2	0.2	13.1	7.6	4.8
Spring	Mean		10.5	100354.5	74.5	10.7	13.7	15.2	572.6	29.6	160503.8	14.1	11.5	3.5	0.1
	Median		10.1	100318.1	75.1	7.3	9.4	10.4	576.3	28.4	163306.2	14.1	8.2	2.7	0.0
	Percentiles	25	5.5	99938.0	67.4	3.4	4.5	4.6	515.1	24.7	149051.6	14.0	5.4	2.4	0.0
		50	10.1	100318.1	75.1	7.3	9.4	10.4	576.3	28.4	163306.2	14.1	8.2	2.7	0.0
		75	15.0	100733.1	81.5	14.5	18.4	20.4	634.1	33.9	174005.8	14.3	14.0	3.1	0.1
	Std. Dev.		6.3	698.0	8.8	11.6	14.2	16.1	77.5	6.5	19294.8	0.3	9.6	4.0	0.2
Summer	Mean		23.9	100120.9	72.1	5.7	7.6	8.1	544.9	44.1	167129.2	14.1	6.2	2.7	0.0
	Median		23.6	100128.8	71.4	5.3	7.2	7.8	546.8	44.5	167383.0	14.1	5.4	2.7	0.0
	Percentiles	25	19.8	99887.9	64.6	3.8	4.9	5.0	481.7	36.8	155592.5	14.0	3.4	2.3	0.0
		50	23.6	100128.8	71.4	5.3	7.2	7.8	546.8	44.5	167383.0	14.1	5.4	2.7	0.0
		75	28.2	100337.1	79.4	7.7	9.9	10.9	606.7	50.8	179297.2	14.3	8.0	3.4	0.1
	Std. Dev.		5.6	362.9	9.2	3.1	3.9	4.4	74.9	10.1	17647.3	0.2	5.1	2.6	0.1
Fall	Mean		11.5	100749.2	78.7	14.8	18.8	20.9	593.9	24.9	188625.0	14.1	9.1	4.0	0.4
	Median		10.6	100797.0	79.8	9.3	12.2	13.3	586.6	22.8	192119.8	14.1	8.2	3.0	0.1
	Percentiles	25	7.1	100321.1	73.0	5.2	7.1	7.6	522.8	20.6	172569.5	14.0	4.9	1.6	0.0
		50	10.6	100797.0	79.8	9.3	12.2	13.3	586.6	22.8	192119.8	14.1	8.2	3.0	0.1
		75	15.2	101275.6	85.4	20.6	25.9	28.9	673.3	27.3	208246.5	14.2	13.0	4.3	0.2
	Std. Dev.		6.1	714.5	8.3	15.4	18.8	21.4	91.0	5.8	28378.6	0.2	6.4	4.4	2.0

### Spearman’s rho correlation analysis

A strong and positive correlation (*r* = 0.880) can be observed in Table 3 between temperature and ozone concentration. This means that ozone levels tend to increase as atmospheric temperature increases. Especially in the summer, seasonal increases in ozone levels occur due to photochemical reactions as the intensity of sunlight increases. Strong negative correlations are observed between humidity and temperature (*r* = -0.558) and ozone (*r* = -0.590), reflecting a significant inverse relationship between the tendency for these parameters to decrease with increasing relative humidity. A slightly positive relationship (*r* = 0.240) was detected between particulate matter concentrations (PM_1_, PM_2.5_, and PM_10_) and pressure. This indicates that atmospheric pressure increases may slightly increase PM concentrations under certain conditions. However, negative correlations between VOC and PM concentrations (*r* ≈ -0.450) suggest possible chemical and physical interactions between the increase of VOCs and the decrease in PM concentrations. The small but statistically significant positive correlation between VOC and CO_2_ (*r* = 0.156) suggests that these two pollutants may have common sources or be affected by similar environmental processes. Correlations between formaldehyde (CH_2_O) and other parameters are weak, indicating that somewhat independent dynamics may drive atmospheric levels of CH_2_O. Ozone shows a strong negative correlation with NO₂ (*r* = -0.518) and CO (*r* = -0.474), indicating that ozone levels tend to decrease as NO₂ and CO levels increase. The correlation between ozone and NO is positive but weak (*r* = 0.068), indicating a slight direct relationship between the two pollutants.

**Table 3:** Correlation analysis of the data.

T (°C)													
P (Pa)	− 0.244**												
RH (%)	− 0.558**	− 0.050**											
PM _1_(µg/m³)	− 0.554**	0.240**	0.359**										
PM_2.5_ (µg/m³)	− 0.555**	0.240**	0.359**	1.000**									
PM_10_ (µg/m³)	− 0.554**	0.240**	0.359**	1.000**	1.000**								
CO _2_ (ppm)	− 0.381**	0.235**	0.483**	0.077**	0.078**	0.077**							
O _3_ (µg/m³)	0.880**	− 0.295**	− 0.590**	− 0.542**	− 0.542**	− 0.542**	− 0.343**						
VOC (ppb)	0.070**	0.055**	0.044**	− 0.450**	− 0.450**	− 0.450**	0.156**	− 0.096**					
CH _2_ O (µg/m³)	0.037**	− 0.033**	-0.02	− 0.047**	− 0.047**	− 0.047**	− 0.025*	0.046**	-0.02				
NO _2_ (µg/m³)	− 0.596**	0.082**	0.319**	0.556**	0.557**	0.557**	0.00	− 0.518**	− 0.272**	− 0.040**			
NO (µg/m³)	0.073**	− 0.031**	− 0.189**	-0.01	-0.01	-0.01	− 0.148**	0.068**	-0.02	0.01	0.145**		
CO (µg/m³)	− 0.525**	0.144**	0.272**	0.552**	0.552**	0.552**	0.00	− 0.474**	− 0.179**	− 0.046**	0.620**	0.039**	
	T (°C)	P (Pa)	RH (%)	PM _1_ (µg/m³)	PM _2.5_ (µg/m³)	PM _10_ (µg/m³)	CO _2_ (ppm)	O _3_ (µg/m³)	VOC	CH _2_ O (µg/m³)	NO _2_ (µg/m³)	NO (µg/m³)	CO (µg/m³)

## Weekday and weekend comparison using independent samples T-Test

In addition to these seasonal changes, a T-Test compared pollutant concentrations between weekdays and weekends (Table 4). According to the results, temperature showed a statistically significant difference with a mean difference of (-0.868 °C) (*p* < 0.001), indicating that weekdays tend to have slightly lower temperatures than weekends. However, pressure did not significantly differ (*p* = 0.264), indicating that atmospheric pressure remains relatively constant regardless of the day. Relative humidity levels were significantly higher on weekdays (*p* = 0.006) and the mean difference was 0.596%. For particulate matter concentrations (PM1, PM2.5 and PM10), no significant difference was observed between weekdays and weekends (*p* > 0.8). CO_2_ levels were significantly higher on weekdays (*p* = 0.045). In contrast, O₃ concentrations did not differ significantly between weekdays and weekends (*p* = 0.912). VOC showed a significant decrease on weekdays compared to weekends (*p* < 0.001). NO₂ and NO levels were significantly higher on weekdays (*p* < 0.001 for both), which may reflect the impact of weekday traffic and industrial activity on these pollutants. Finally, CO levels were significantly lower on weekdays than weekends (*p* < 0.001).

**Table 4:** Independent two sample T-Test results for weekday and weekend air quality parameters.

Parameters	t	df	Sig. (2-tailed)	Mean Difference	Std. Error Difference
Temperature (°C)	-3.856	8308	0.000	-0.868	0.225
Pressure (Pa)	-1.117	8308	0.264	-19.750	17.690
Humidity (%)	2.759	8308	0.006	0.596	0.216
PM_1_ (µg/m³)	0.195	8308	0.846	0.075	0.384
PM_2.5_ (µg/m³)	0.205	8308	0.838	0.096	0.470
PM_10_ (µg/m³)	0.222	8308	0.824	0.119	0.535
CO_2_ (ppm)	2.003	8308	0.045	3.873	1.934
O_3_ (µg/m³)	-0.111	8308	0.912	-0.029	0.261
VOC (ppb)	-3.833	8308	0.000	-277.020	72.670
CH_2_O (µg/m³)	0.150	8308	0.880	0.001	0.006
NO_2_ (µg/m³)	11.816	8308	0.000	3.000	0.254
NO (µg/m³)	12.404	8308	0.000	1.511	0.122
CO (µg/m³)	-4.481	8308	0.000	-0.287	0.064

During COVID-19, higher weekend ozone effects were reported for small and medium-sized cities in Italy and the USA when it was recorded a simultaneous reduction of NO_x_ and VOC, not for a NO_x_reduction alone^71,72^. After the pandemic lockdowns, it was noticed that the weekend ozone effect decreased in small and mid-sized cities. In another research paper, the ozone weekend effect was connected to the ratio PM2.5/PM10^73^. In all four studied cities, a positive weekend effect was recorded for this ratio during the pandemic, but this intensity suffered significant reductions after the return to normal activities. The restrictions imposed by the Romanian authorities during the COVID-19 pandemic were less severe during the studied period (December 10, 2020 - December 9, 2021) in this study than the state of emergency (March 16, 2020 - May 15, 2020).

The current study shows that the weekend ozone effect is very low in Craiova (weekend-weekday difference < 5%, according to the classification made by Sicard et al^74^.. During the weekend, there is a NO_x_ reduction, accompanied by an increase in VOC, and the PM concentration values remain constant. During the weekends, the temperature is slightly higher than on weekdays, and the relative humidity is lower, so ozone levels do not change significantly over the weekend. Meteorological factors significantly impact ozone concentrations more than daily human activities, such as traffic, industrial activities, and the heating system of houses based on fossil fuels or wood.

### Ozone thresholds analysis

Following established guidelines for air quality standards, the ozone (O_3_) concentration data analysis focused on identifying exceedance days, which are occasions when observed levels exceed the recommended threshold limits. The dataset generated from the measured data was processed by scripting using Python 3.11 to calculate 8-hour rolling average concentrations each day, a critical metric for assessing ozone exposure levels^75,76^.

The World Health Organization (WHO) recommends that ozone concentrations for long-term exposure should not exceed 100 µg/m^3^, calculated as the 99th percentile for peak season, defined as 3–4 exceedance days per year. And long-term exceedance targets of 60 µg/m^3^ for 8-hour intervals. On the other hand, the European Union (EU) Air Quality Directive (2008/50/EC) sets the threshold at 120 µg/m^3^ on a maximum daily 8-hour average, allowing up to 25 exceedance days per year as an average over three years. According to computation, the 8-hour rolling average of ozone concentration O_3_ (t) is used to identify exceedance days according to WHO and EU guidelines within a year using Eq. 1:

1 $$\overline{O_3}\left(t\right)=\frac1N\sum_{i=0}^{N-1}O_3\left(t-i\right),$$

where *t* represents the 8-hour rolling average of ozone concentration at a time.

According to the WHO recommendations, long-term exposure exceedance occurs at over 60 µg/m^3^ for any 8 h within a day, and short-term exposure exceedance occurs at over 100 µg/m^3^ for any 8 h within a day. According to the EU directive, long-term exposure exceedance occurs at over 120 µg/m^3^ for over 25 days per year. Based on the results calculated using this code, 13 samples were observed where the WHO threshold for long-term exposure (60 µg/m^3^) was exceeded based on the 1-year data set. These samples are not evenly distributed throughout the year but are determined by specific times of day, possibly reflecting the temporal variability of ozone levels affected by daily emission patterns and meteorological conditions. The limit value was exceeded by three days in June, seven days in July, and three days in August. Notably, there were no examples where the WHO recommended threshold for peak season exposure (100 µg/m^3^ averaged over 8 h) was exceeded. Similarly, the data set revealed that the target value of the EU Directive was not exceeded (maximum daily 8-hour average of 120 µg/m^3^). However, especially for the Ozone, 471,619 data were obtained within a year, and out of these data, 12,948 were above 60 µg/m^3^, 25 were above 100 µg/m^3^, and 10 were above 120 µg/m^3^.

### Multiple linear regression analysis

Table 5 presents a regression analysis that evaluates the impact of various environmental factors on atmospheric ozone (O_3_) concentrations and quantifies the extent to which these factors contribute. The R coefficient (0.913) measures how well the independent variables predict the dependent variable. R squared (R² = 0.833) indicates that the independent variables explain 83.3% of the variance in ozone concentrations.

**Table 5:** Multiple linear regression analysis.

R	R^2^	Adjusted R^2^	Std. Error of the Estimate
0.913^a^	0.834	0.833	4.388

^a^Predictors: (Constant), Temperature, Relative Humidity, Pressure, CO_2_, PM_1_, PM_2.5_, PM_10_, VOC, CH_2_O, NO_2_, NO, CO.

Table 6 presents ANOVA (Analysis of Variance) test results on whether the regression model is statistically significant. In this case, the regression model shows, based on the F-test, that there is a statistically significant relationship between predicted ozone concentrations and actual measurements (F = 3778.081, *p* < 0.001). A large portion of the total variance given by the model is explained by the regression (R² = 0.833), indicating that the model largely explains the variation in ozone concentrations of the independent variables.

**Table 6:** ANOVA test results.

	Sum of Squares	df	Mean Square	F	Sig.
Regression	800251.785	11	72750.162	3778.081^a^	0.000^b^
Residual	159765.805	8308	19.256		

^a^Dependent Variable: O_3_.

^b^Predictors: (Constant), Temperature, Humidity, Pressure, CO_2_, PM_1_, PM_2.5_, PM_10_, VOC, CH_2_O, NO_2_, NO, CO.

Additionally, Table 7 shows each independent variable’s estimated effect on ozone concentration. The constant term (Constant) is the estimated ozone concentration (135.458 µg/m³) when all other variables have zero values. The temperature coefficient (Beta = 0.848, t = 123.909, *p* < 0.001) shows that most of the changes in ozone levels are explained by temperature. Pressure and humidity also significantly affect ozone concentrations, both having a negative coefficient and decreasing ozone levels. The positive coefficient of _PM10_ (Beta = 1.330) indicates that this size of particulate matter has an increasing effect on ozone levels. In contrast, the negative coefficient of PM_1_ (Beta = -1.862) indicates that these particle sizes reduce ozone concentration. The exclusion of PM2.5 from the model is likely due to multicollinearity issues, as this variable strongly correlated with either PM1 or PM10, leading to redundancy in the model. Although the coefficients for VOC and CO_2_ are lower, both contribute to the significance of the model. While a negative relationship was found for VOC (Beta = -0.101), a positive relationship was found for CO_2_ (Beta = 0.040). NO_2_ (Beta = 0.060, t = 9.741, *p* < 0.001) is positively correlated with ozone, indicating that an increase in NO2 levels contributes to an increase in ozone concentration. On the other hand, NO (Beta = -0.055, t = -11.015, *p* < 0.001) has a negative correlation, indicating that higher NO levels reduce ozone concentrations. Finally, CO (Beta = 0.006, t = 1.272, *p* = 0.203) has a positive coefficient, while its effect is not statistically significant, indicating that CO has little or no significant effect on ozone levels in this model.

**Table 7:** Coefficient results.

	Unstandardised Coefficients		Standardised Coefficients	t-statistics	Sig.
	B	Std. Error	Beta		
(Constant)	135.458	8.354		16.214	0.000
T (°C)	0.983	0.008	0.848	123.909	0.000
P (Pa)	-0.001	0.000	-0.071	-13.725	0.000
RH (%)	-0.145	0.008	-0.120	-18.621	0.000
PM_1_ (µg/m³)	-1.862	0.250	-2.739	-7.439	0.000
PM_10_ (µg/m³)	1.330	0.180	2.727	7.402	0.000
CO_2_ (ppm)	0.005	0.001	0.040	6.826	0.000
VOC (µg/m³)	-3.637 × 10^−5^	0.000	-0.101	-18.020	0.000
CH_2_ O (ppb)	0.147	0.208	0.003	0.705	0.481
NO_2_ (µg/m³)	0.061	0.006	0.060	9.741	0.000
NO (µg/m³)	-0.116	0.011	-0.055	-11.015	0.000
CO (µg/m³)	0.024	0.019	0.006	1.272	0.203

^a^Dependent Variable: O_3._

Based on a dataset that spans 1994–1996, Pont and Fontan^77^ showed the importance of the advection phenomena of ozone in four big French cities: Marseilles, Lyon, Paris, Strasbourg, and Toulouse. They found a difference of 20% between the highest values of O_3_concentrations measured on weekdays and during the weekend, whereas traffic levels vary by more than 40% between Fridays and Sundays. Wang et al^78^. showed that in Beijing, weekend hourly O_3_ concentrations were higher than weekday ozone concentrations between 11:00 and 24:00, suggesting a significant weekend effect because weekend NO inhibition is weaker than weekday NO inhibition during the O_3_ formation phase. Craiova does not belong to the category of big cities, and the traffic variation is not as high as in French or Chinese cities.

In a complex study conducted in Hong Kong, Zang et al^79^. observed that O_3_ levels rise in the late morning. O_3_ peaks near noon and starts to decrease in the early afternoon. This behaviour is attributed to high NO concentrations, intense sunlight, and the transport of air masses with different chemical characteristics. Another observation for Hong Kong is that VOC limits O_3_ production, while high nitric oxide (NO) concentrations suppress O_3_ concentration. In Craiova, like in Hong Kong, O_3_ formation is limited by VOC, and high NO_x_ concentrations suppress O_3_ production.

Nap et al^80,81^. revealed the importance of meteorological factors and other gases on ozone concentrations and emphasised the importance of statistical analysis in estimating ozone concentrations. These studies measured hourly variables like O_3_, NO₂, NO, temperature, relative humidity, and wind speed data. A study was conducted in an industrial area of Terengganu, Malaysia^81^. The authors developed three different MLR models for predicting ozone concentrations, with the most accurate model achieving an R^2^ of 0.792. This study highlighted the significant impact of meteorological conditions and other gaseous pollutants on ozone levels. Modelling was performed using the MLR method to study the temporal distribution of PM concentrations and O_3_pollutants in Myanmar^48^. It was noticed that PM_2.5_ and PM_10_ concentrations exceeded the WHO-acceptable threshold levels, with significant peaks in air pollutants during summer and winter. As in the current study, O_3_concentrations showed a significant negative correlation with all pollutants and a significant positive correlation with ambient temperature. The study from Hyderabad^82^ focused on the seasonal prediction of ground-level ozone concentrations using MLR models. The study found that ozone was positively correlated with temperature, solar radiation, and wind speed and negatively correlated with NO_x_, CO, and relative humidity. The models achieved adjusted R^2^values ranging from 0.6 to 0.9, depending on the season. In a study developed in the North China Plain^83^, the authors investigated the sensitivity of PM_2.5_ and O_3_ pollution events to four selected meteorological factors (wind speed, temperature, water vapours mixing ratio, and planetary boundary layer height) during two heavy pollution events. According to the results, significant spatial and seasonal variations in PM_2.5_ and O_3_ concentrations were observed due to meteorological factors. All these authors showed that the MLR model represented a solid and appropriate tool for O_3_ prediction and highlighted the variation of O_3_ level with the season of pollution and local conditions.

Su et al^84^. showed that high ozone concentrations are associated with China’s urban and downwind air masses. They found that VOC and NO_x_are the key drivers of ozone formation. However, this study observed that ozone was more related to temperature than VOC, as in the present study. In a study developed in Agadir, Morocco^85^, using a mobile monitoring station, forward regression analysis was applied to predict daily ozone concentrations based on meteorological parameters and primary pollutants. The model achieved an R^2^ of 0.886, indicating a strong fit, with significant predictors including temperature, wind speed, NO_2_, and CO. The results underscored the importance of these factors in influencing ozone levels. Duarte et al^86^. and Orellano et al^87^. concluded significant spatial and temporal differences in PM and O_3_ concentrations. In addition, high temperatures and low humidity conditions were generally associated with higher O_3_ levels due to enhanced photochemical reactions. The correlation analysis between atmospheric pollutants and meteorological variables revealed essential insights. PM_10_ and PM_2.5_exhibit a strong positive correlation, indicating similar sources or formation mechanisms. This contradicts our results (negative correlation with all pollutant concentrations). Local and seasonal variations significantly affect pollution levels, which agrees with our results. The authors of a study developed in India^43^, examined 8-hour averages of ozone concentrations and the effects of ozone on human health, the environment and building materials. They emphasised that in this country, ozone levels reach maximum unhealthy hours during winter, which disagrees with our findings; for the current study from Craiova, Romania, the highest VOC level is recorded during winter when the temperatures are low and do not favour ozone formation reactions. From this perspective, the temperature influences the ozone level more than the VOC.

A study from Warsaw^88^highlighted the role of local photochemistry in ozone formation during heat waves when all meteorological conditions brought their contribution. Craiova is highly likely to experience heat waves for 2–5 days, in exceptional situations of 18–20 days^89^, when ozone levels increase significantly from June to August. These examples highlight the significant role of meteorological factors (high temperature, high number of sun brightness hours, low relative humidity, low wind speed) and nitrogen oxides (from traffic) in ozone formation. In a study conducted in the Yangtze River Delta from 2016 to 2023^90^, although NO2, CO, and PM2.5 decreased, the ozone level exhibited fluctuating patterns. In the long term, variations of ozone levels from emissions were identified as the predominant factor shaping annual fluctuations in ambient ozone. The effect of weather conditions on ozone concentration is essential in the short term. It can increase or decrease the effectiveness of precursor emission controls the efficacy of precursor emission control in diminishing ozone concentrations. Differentiating the contributions of meteorological conditions and emission changes to ozone is important for assessing the effectiveness of control measures on past datasets and developing new future approaches. In order to achieve such a goal in Craiova, it is necessary to use several monitoring stations evenly distributed throughout the city to measure more meteorological parameters than in the present study and more types of emissions.

In research on the ozone level in Ciuc Depression (Romania)^91^, based on a one-year dataset related to solar radiation, temperature, NO_x_ and ozone, the authors noticed an accentuated ozone formation with high static stability during winter and spring. In the summer, high ozone concentrations were observed when the intense solar radiation, high temperatures and longer day lengths stimulated photochemistry. The climate in Ciuc Depression is s characterised by high frequency and long persistence of nocturnal and winter temperature inversions. In Craiova, things are very different during winter. However, ozone concentration increases during spring, touches the maximum during summer and decreases during autumn. These differences emphasise the importance of understanding ozone formation locally. Traffic and industrial activities raise NO, NO_2_ and VOC emissions on weekdays. On weekends, traffic and industrial activities decrease, leading to a decrease in NO and NO2 concentrations. It is well-known that the ratio between nitrogen oxides and VOC is very important in ground-level ozone production, which is formed by photochemical reactions between nitrogen oxides and VOC in sunlight. On weekdays, high nitrogen oxide concentrations can react with existing ozone, reducing ground ozone concentrations. During the weekends, the variation of meteorological parameters can favour ozone accumulation, but as they are not constant, they influence ozone concentrations differently from weekend to weekend.

## Conclusion

1. The field measurements provided the dataset, which included various air quality indices such as particulate matter (PM1, PM2.5, PM10), CO2, VOC, CH2O, NO2, NO, CO and O3, and meteorological parameters (T, P, RH). The results from statistical analyses emphasize significant seasonal variations in ozone concentrations in Craiova, Romania. The SPSS Data Analysis results showed that ozone levels were lowest in late autumn and winter and highest in late spring and summer, which aligns with the most important part of the previous studies. In addition, PM10 exhibited a significant correlation with ozone, indicating that particulate matter can influence ozone levels, while VOC showed a negative correlation with O3 concentrations. The relationship between ozone and NO2 was positive, highlighting the role of NO2 in O3 production, whereas NO had a negative correlation due to its role in ozone depletion. Carbon monoxide (CO) did not show a statistically significant effect on ozone levels in this analysis.
2. According to the T-Test, significant differences were observed in temperature, relative humidity, CO2, VOC, NO2, NO and CO levels between weekdays and weekends. These results show that some pollutant levels (NO2, NO) decreased during weekends, especially due to decreased traffic and industrial activities. However, increases in other pollutants (such as VOC and CO). No significant differences were observed between weekdays and weekends for particulate matter concentrations (PM1, PM2.5 and PM10). Ozone levels did not show significant differences between weekdays and weekends. This shows that meteorological factors had a more significant impact on weekend ozone effect than anthropocentric emissions. It is known that a simultaneous reduction of NOx and VOC is more effective than a NOx reduction on the weekend ozone effect.
3. Considering the ozone limit values from the WHO and EU Air Quality Directive, the number of days the limits exceeded was determined. The results show that the 60 µg/m3 limit value exceeds 13 days annually, especially during summer. However, especially for the ozone, 471,619 data were obtained within a year; 12,948 were above 60 µg/m3, 25 were above 100 µg/m3, and 10 were above 120 µg/m3.
4. The relationship between ozone concentrations, meteorological factors, and other pollutants is complex. The findings highlight the importance of understanding this relation locally before developing effective air pollution reduction strategies and protecting public health. This research offers valuable data on seasonal O3 variations and their relation to other pollutants in Craiova. The insights gained from this study are primarily focused on understanding the local environmental conditions and pollutant dynamics. It lays the groundwork for more detailed investigations that could potentially guide such efforts.
5. This research underscores the need for continuous monitoring and analysis of air quality in urban areas, particularly in the face of seasonal variations and their impact on pollutant levels. Future studies should integrate a wider range of pollutants from a larger number of sensors evenly distributed in Craiova, wind speed and direction and advanced modelling techniques for a better understanding of the weekend ozone effect.

## Electronic supplementary material

Below is the link to the electronic supplementary material.

Supplementary Material 1

## Data Availability

Data supporting the results of the study can be accessed upon reasonable request from the corresponding author.
