# Supplementary material for: Observational study of ground-level ozone and climatic factors in Craiova, Romania, based on one-year high-resolution data
Source: Sci Rep. 2024 Nov 5;14:26733. doi: 10.1038/s41598-024-77989-0 (PMC11538392; doi:10.1038/s41598-024-77989-0)
Supplement: Supplementary file 1 — Supplementary Material 1 [file 41598_2024_77989_MOESM1_ESM.docx]

# Appendix A

Technical specifications of the sensor used in this work:

The monitoring system uRADmonitor A3 has the ID 820002C3, weighs 170g, and has small sizes (110 mm length x 65 mm width x 25 mm depth). The monitor system collected the dataset used in this study in the first year after its installation (2020-2021). This detail is essential because the lifespan of the electrochemical sensor that measures O3 concentration is two years. Moreover, in the second and third years, the values for O3 concentration were significantly lower than in the first year. For this reason, the authors decided to use only the dataset from the first year after monitor’s installation.


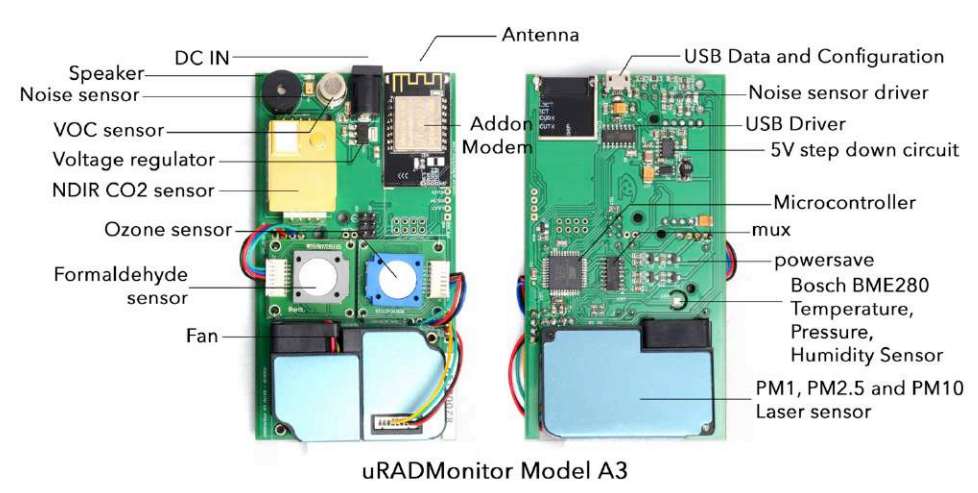


Figure 1. uRADMonitor A3 motherboard, top and bottom, hardware version HW108 (Source: https://www.uradmonitor.com/wp-content/uploads/2021/08/a3_datasheet_v109_en_compressed.pdf)

Microelectromechanical systems (MEMs) measure the meteorological parameters:

- air temperature T (measuring range is between -40 °C and +85 °C, 0.5 °C resolution, ± 1° accuracy),
- air pressure P (range 300 - 1100 hPa, ± 0.25 % accuracy),
- relative humidity RH (1% RH resolution, ± 2%accuracy).

The PM sensor (PM1, PM2.5, PM10) has a measuring interval between 0 and 1000 µg/m³, a resolution of 1 µg/m3, and a correlation coefficient R=0.99 to reference gravimetric sampler Sven Leckel LVS3 determined by ISO17025. The dioxide of carbon concentration is measured by a non-dispersive infrared sensor(measuring interval is 400 -5000 ppm, 1 ppm resolution and ±5% accuracy).

Two electrochemical sensors track:

- formaldehyde (range 0 - 5 ppm, 10 ppb resolution and ±5% accuracy, two years lifespan) and
- Ozone(measuring interval 0 - 10 ppm, 10 ppb resolution and ±5% accuracy, two years lifespan).

A metal-oxide sensor determines volatile organic compounds (range 10 - 1000 ppm estimated for alcohol, ±5% accuracy).

An analogic sensor measures the noise level (30 - 130 dB, 1 dB resolution, ±10% accuracy).

Before selling each sensor, the manufacturer benchmarks it against a reference sensor. In this process, the calibration graphs are plotted. For the monitor A3, the calibration is embedded in the sensor software (according to the recommendations of those three independent laboratories).

Laser scattering is the method used by uRADMonitor A3 for measuring particulate matter concentrations. This method differs from the gravimetric method (used by the Romanian National Air Quality Monitoring Network). If the gravimetric method is based on the weight difference of filters pre- and post-sampling, a pulse of coherent infrared light shines through a cavity for the laser scattering method. When the laser beam reaches a particle, it scatters the laser light. A PIN photodiode, which is located in the chamber, detects the light signal. There is a proportional relation between the amplitude of the recorded scattered signal and the particle size, and the number of events is correlated with the mass concentration.”

Sources:

*https://www.uradmonitor.com/wp-content/uploads/2019/04/INCD-ECOIND_final_report_Magnasci_uRADMonitor_A3_HW107.pdf (accessed on July 22, 2024)

**https://www.uradmonitor.com/wp-content/uploads/2019/04/uradmonitor_a3-field-evaluatione840a0efc2b66f27bf6fff00004a91a9.pdf (accessed on July 22, 2024)

***https://www.uradmonitor.com/wp-content/uploads/2021/08/a3_datasheet_v109_en_compressed.pdf (accessed on July 22, 2024)
